# Predictive factors of urinary tract infections among the oldest old in the general population. a population-based prospective follow-up study

**DOI:** 10.1186/1741-7015-9-57

**Published:** 2011-05-16

**Authors:** Monique AA Caljouw, Wendy PJ den Elzen, Herman JM Cools, Jacobijn Gussekloo

**Affiliations:** 1 Department of Public Health and Primary Care, Leiden University Medical Center, Netherlands

## Abstract

**Background:**

Urinary tract infections (UTI) are common among the oldest old and may lead to a few days of illness, delirium or even to death. We studied the incidence and predictive factors of UTI among the oldest old in the general population.

**Methods:**

The Leiden 85-plus Study is a population-based prospective follow-up study of 86-year-old subjects in Leiden, The Netherlands. Information on the diagnosis of UTI was obtained annually during four years of follow-up from the medical records and interviews of treating physicians. A total of 157 men and 322 women aged 86 years participated in the study. Possible predictive factors were collected at baseline, including history of UTI between the age of 85 and 86 years, aspects of functioning (cognitive impairment (Mini-Mental State Examination (MMSE) < 19), presence of depressive symptoms (Geriatric Depression Scale (GDS) > 4), disability in activities of daily living (ADL)), and co-morbidities.

**Results:**

The incidence of UTI from age 86 through 90 years was 11.2 (95% confidence interval (CI) 9.4, 13.1) per 100 person-years at risk. Multivariate analysis showed that history of UTI between the age of 85 and 86 years (hazard ratio (HR) 3.4 (95% CI 2.4, 5.0)), impaired cognitive function (HR 1.9 (95% CI 1.3, 2.9)), disability in daily living (HR 1.7 (95% CI 1.1, 2.5)) and urine incontinence (HR 1.5 (95% CI 1.0, 2.1)) were independent predictors of an increased incidence of UTI from age 86 onwards.

**Conclusions:**

Within the oldest old, a history of UTI between the age of 85 and 86 years, cognitive impairment, ADL disability and urine incontinence are independent predictors of developing UTI. These predictive factors could be used to target preventive measures to the oldest old at high risk of UTI.

## Background

Urinary tract infections (UTIs) are common in the very elderly and account for nearly 25% of all infections [1,2]. The incidence of UTI increases with age in both men and women [3-5], and increases from 12 to 29 per 100 person-years at risk in community-dwelling elderly populations [5,6] to 44 to 58 per 100 residents per year at risk in long term care facilities [7,8]. These UTI are often complicated, involving the presence of structural or functional abnormalities of the genitourinary tract [9]. Especially in vulnerable older persons living in long term care facilities, UTIs more often have serious consequences such as delirium, dehydration, urosepsis, hospitalisation, or even death [10,11].

Several strategies to prevent UTI have been developed, such as treatment of those at high risk with low-dose, long-term antibiotics [12,13], oestrogens [14] and cranberry products [12,15]. These strategies have been shown to be effective in preventing UTI in younger women with recurrent UTI [13-15], but not yet in vulnerable older people. Preventive strategies are best applied to those at risk; however, factors associated with UTI in ambulatory older patients in the community setting have not been described.

Previous studies have shown that increasing age [4,16], diabetes mellitus [17,18], stroke [19], urine incontinence [1,20,21], prior history of UTI [1,20], urogenital surgery [1,20], and impaired functional and cognitive status [16,20] predict the development of UTI among older individuals. However, these studies investigated predictors of UTI in specific patient groups, such as hospitalised patients and patients residing in long-term care facilities and did not include older individuals in the general population. In addition, these studies used different methods to identify UTI. It is generally accepted that diagnosing symptomatic UTI in older persons is complicated due to factors like difficult doctor-patient communication, chronical genito-urinary symptoms, and a high frequency of positive urine cultures due to bacteriuria without complaints [22].

To target preventive strategies against UTIs in older individuals, those with highest risk to develop UTI have to be identified. The purpose of this study was to determine the incidence and predictive factors of UTI among the oldest old in the general population.

## Methods

### Setting and study population

The Leiden 85-plus Study is an observational population-based prospective study of 85-year-old inhabitants of Leiden, The Netherlands. Between September 1997 and September 1999, all inhabitants of Leiden who reached the age of 85 years were invited to participate in the study. There were no selection criteria concerning health or demographic characteristics. The medical ethics committee of the Leiden University Medical Center approved the study. All participants gave informed consent for the whole study including the use of data from their medical records for additional analysis, following explanation of the study requirements and assurance of confidentiality and anonymity. For participants with severe cognitive impairment, a guardian gave informed consent.

The present study was conducted within the framework of the Leiden 85-plus Study. We started follow-up for four years at age 86 years to allow us to study 'history of UTI between the age of 85 and 86 years' as a possible predictor. In this study, 479 participants aged 86 years were included. Participants were revisited annually until the age of 90 years. All participants were visited at their place of residence where face-to-face interviews were conducted, cognitive testing was performed, information on socio-demographic characteristics and disabilities in daily living was obtained, and a venous blood sample was taken.

### Urinary tract infection

The endpoint of this study was the development of the first UTI from age 86 through 90 years. This endpoint was considered present when treating physicians diagnosed UTI based on signs and symptoms and urine analysis.

The endpoint was also reached when a participant during follow-up died from UTI. General practitioners and elderly care physicians were interviewed annually to gather clinical information. Each year data were gathered about the development of clinical diagnosed UTI during the preceding year from clinician interviews and records. Mortality data were obtained from the municipality. Specific data on causes of death were obtained from Statistics Netherlands, according to the International Classification of Diseases and Related Disorders, 10^th ^revision (ICD-10), including UTI (ICD-10 code N39.0) [23].

### Selection of potentially predictive factors

Through an extensive search of scientific literature factors that are potentially predictive for UTI in older individuals were identified and selected for the study, within the domains socio-demographic factors, functioning, co-morbidities and renal functioning.

#### Socio-demographic factors

During baseline interviews, a research nurse collected information about the participants' residency, income, level of education, body mass index and smoking habits.

#### Functioning

To assess cognitive function, the Mini-Mental State Examination (MMSE) was administered. Severe cognitive impairment was defined as a MMSE score below 19 points [24,25]. The Geriatric Depression Scale-15 (GDS-15) was performed to determine the presence of depressive symptoms. The presence of depressive symptoms was defined as a GDS-15 score above four points. The GDS-15 could only be administered in participants with an MMSE score above 19 points [26,27]. Disability in basic activities of daily living (ADL) was determined using the Groningen Activity Restriction Scale (GARS) [28] and defined as being unable to do any one of the following nine ADL: independently: walk inside, get out of bed, get into and out of a chair, use the toilet, wash hands and face, wash body, dress and undress, eat and drink, and make breakfast [29]. The GARS-items were dichotomized. Participants were grouped into those who had no difficulty with GARS-items (score 1) and those who had difficulty or were unable to perform the GARS-items independently (score 2, 3 and 4). The total GARS score was calculated by adding the total scores of the nine items of the GARS and than dichotomized into score 9 (independently) and scores > 9 (difficulty or unable to perform independently). All questionnaires were validated in Dutch.

#### Co-morbidities

Information on participants' medical history was obtained by standardised interviews with their treating general practitioner or elderly care physician and by examination of pharmacy records. We obtained clinical information on the presence of diabetes mellitus, stroke and cancer as well as information on unintentional loss of faeces and/or urine. Diagnosis of incontinence was ascertained by the general practitioner or elderly care physician. For males, complaints of Lower Urinary Tract symptoms (LUTS) caused by benign prostatic hyperplasia (BPH) were measured with the International Prostate Symptom Score (IPSS). The IPSS is an eight-question written screening tool to rapidly diagnose BPH, track the symptoms of BPH and suggest management of the symptoms of BPH [30]. The severity of urine incontinence was obtained by the PRAFAB-Questionnaire, which combines objective and subjective aspects of the severity of urinary incontinence [31-33]. PRAFAB stands for Protection (the use of pads), Amount of urine loss, Frequency of urine loss, Adjustment of behaviour due to symptoms, and Body (or self) image as a result of the stress urine loss symptoms.

Serum creatinine concentration was measured automatically according to the Jaffe method (Hitachi 747; Hitachi, Tokyo, Japan). Creatinine clearance was calculated from serum creatinine concentration and body weight, using the Cockcroft-Gault formula [34]. Low creatinine clearance was defined as a creatinine clearance below 30 mL/minute. For unknown disease, we measured C-reactive protein (CRP) levels with a fully automated Hitachi 911 analysis system. CRP levels above 5 mg/L were considered elevated CRP levels [35].

### Data analyses

The incidence of UTI from age 86 years onwards was calculated during four years of follow-up (until age 90 years), using the life-table method. The number of first time UTI was assigned to the numerator and the observed person-years at risk were assigned to the denominator. The observed person-years at risk were counted from age 86 to the censor date (age 90 years), to date of death, or to date of first UTI.

The association between predictive factors and the occurrence of UTI in participants from age 86 years onwards was investigated with Cox proportional hazards models. Those factors predicting UTI in the univariate Cox regression analysis with a *P-*value < 0.10 were included in a multivariate Cox regression analysis. Since the GDS-15 could only be administered to participants without cognitive impairment (MMSE ≥ 19 points, n = 393), the variable 'depressive symptoms' was not included in the multivariate analysis. The relative contribution of the different predictive factors to the occurrence of UTI was determined by calculating the Population Attributable Risk (PAR), which combines the relative risk and the prevalence of the different predictive factors. Data analyses were performed using SPSS for Windows, version 16.0 (SPSS Inc, Chicago, USA).

## Results

### Study population

Between September 1997 and September 1999, 705 participants were eligible for participation in the Leiden 85-plus Study. Ninety-two participants refused to participate and 14 participants died before enrolment, resulting in a study population of 599 participants (response rate of 87%) [36]. For the present study, 72 participants for whom valid clinical information about UTI at age 86 was missing were excluded. Forty-eight participants died before the age of 86, resulting in a study population of 479 persons (response rate of 80.0% of the study population).

Table 1 shows the baseline characteristics of the study population at age 86 years (n = 479). Two-thirds of the population was female and 22% of the participants were institutionalised in long-term care facilities. Restriction in ADL was registered in more than 59% of the participants and 19% had an MMSE-score below 19 points. A total of 44% of the participants reported urine incontinence and in almost 20% of the participants incontinence was diagnosed by the general practitioner or elderly care physician. A total of 15% of the participants had a history of UTI between the age of 85 and 86 years. Additional analysis showed that 8.3% of men and 18.3% of women had a history of UTI between the ages of 85 and 86 years (chi-square 8.3; df = 1; *P *= 0.004).

**Table 1:** Risk of UTI from age 86 years onwards depending on socio-demographic, functional and medical baseline characteristics (n = 479)

	Index group N (%)	Incidence in index group, per 100 py (95% CI)	Incidence in reference group*, per 100 py (95% CI)	HR (95%CI)	*P-*value
*Socio-demographic factors*					
Female	322 (67.2)	12.8 (10.4, 15.2)	7.8 (5.1,10.6)	1.7 (1.1, 2.5)	0.012
Long-term care facility	107 (22.3)	23.5 (16.6, 25.6)	9.1 (7.3, 10.9)	2.4 (1.7, 3.4)	< 0.001
Low income	238 (49.7)	11.7 (9.1, 14.4)	10.5 (7.9, 13.1)	1.1 (0.8, 1.6)	0.547
Primary school only	296 (61.8)	12.8 (10.2, 15.3)	8.9 (6.1, 11.3)	1.5 (1.0, 2.1)	0.044
Smoking (current)	70 (14.6)	12.8 (7.3, 18.3)	11.0 (9.0, 12.9)	1.1 (0.7, 1.8)	0.618
Body Mass index ≥27	212 (47.2)	11.1 (8.4, 13.9)	9.9 (7.5, 12.4)	1.1 (0.8, 1.6)	0.535
*Functioning*					
Severe cognitive impairment (MMSE < 19)	90 (19.0)	27.0 (18.6, 35.3)	9.0 (7.2, 10.8)	2.7 (1.9, 3.9)	< 0.001
Depressive symptoms (GDS-15 > 4)	58 (14.8)	8.8 (4.0, 13.5)	9.1 (7.2, 10.9)	1.0 (0.5, 1.7)	0.897
Disability in daily living †	283 (59.2)	16.2 (13.0, 19.4)	6.3 (4.3, 8.3)	2.4 (1.6, 3.5)	< 0.001
*Co-morbidities *					
Diabetes mellitus	76 (15.9)	10.6 (5.8, 15.4)	10.4 (8.4, 12.3)	1.0 (0.6, 1.6)	0.963
Stroke	60 (12.6)	20.8 (12.5, 29.1)	10.2 (8.3, 12.0)	1.9 (1.2, 3.0)	0.004
Cancer	97 (20.6)	10.0 (6.0, 14.0)	11.3 (9.2, 13.4)	1.1 (0.7, 1.8)	0.589
Benign Prostatic Hyperplasia (IPSS score ≥8)	53 (38.1)	8.4 (3.6, 13.2)	6.3 (3.0, 9.6)	1.4 (0.7, 3.1)	0.376
UTI between the ages of 85 and 86 years	72 (15.0)	37.7 (26.5, 48.8)	8.5 (6.8, 10.2	4.1 (2.9, 5.9)	< 0.001
Unintentional loss of faeces	65 (13.6)	32.2 (21.2, 35.6)	9.1 (7.4, 10.9)	3.2 (2.2, 4.8)	< 0.001
Self-reported urine incontinence	212 (44.3)	16.1 (12.5, 19.6)	7.5 (5.5, 9.5)	2.0 (1.4, 2.9)	< 0.001
Medical diagnosis incontinence	95 (19.8)	16.1 (10.5, 21.7)	10.3 (8.4, 12.3)	1.5 (1.0, 2.2)	0.054
PRAFAB score ≥11	100 (81.3)	12.3 (8.0, 16.6)	12.0 (3.1, 21.0)	1.0 (0.4, 2.3)	0.987
PRAFAB: Pad use	91 (74.0)	3.6 (2.5, 5.2)	3.2 (1.7, 6.0)	1.2 (0.6, 2.5)	0.680
Creatinine clearance < 30 mL/min	43 (9.5)	12.1 (5.0, 19.3)	10.6 (8.7, 12.5)	0.9 (0.5, 1.7)	0.794
CRP > 5 mg/L	159 (34.0)	12.9 (9.3, 16.6)	10.1 (8.0, 12.2)	1.2 (0.9, 1.8)	0.222

py = person-years; CI = confidence interval; HR = hazard ratio; CRP = C-reactive protein; GDS-15 = 'Geriatric Depression Scale' with 15 items (only administered to participants with MMSE ≥19 (n = 393)); IPSS = 'International Prostate Symptom Score' (only administered to male participants with MMSE-score ≥19 (n = 139)); MMSE = Mini-Mental State Examination; PRAFAB = five-item questionnaire score incontinence (only administered to participants with MMSE-score ≥19 (n = 123)); UTI = urinary tract infection.

* Definition of reference groups: males, living independently, pension or other extra income, additional education after primary school, not smoking, BMI < 27, MMSE ≥19, GDS-15 ≤4, no disability in daily living, no diabetes, no stroke, IPSS score < 8, no UTI between the ages of 85 and 86 years, no history of cancer, no unintentional loss of faeces, no urine incontinence, no diagnosis incontinence GP/NP, PRAFAB < 8, creatinine clearance ≥30 mL/min, CRP ≤5 mg/L.

† Disability in daily living = unable to do any one of the nine basic activities of daily living independently, according to the Groningen Activity Restriction Scale.

### Incidence of urinary tract infections

In four years of follow-up we observed 140 first episodes of UTI during 1,246 person-years (py) at risk. The overall incidence of UTI was 11.2 (95% CI 9.4, 13.1) per 100 py at risk. Ninety-two participants had recurrent UTI (15.6% of the total population and 47.4% of the participants with UTIs). On average 6.5% (per year range 5.7, 7.3) of all participants experienced two or more UTIs per year during follow-up, of which 45.6% (per year range 40.0, 54.7) had more than one infection per year. A total of 246 participants died during follow-up, of whom seven participants died from UTI according to CBS data. The incidence of UTI was 12.8 (95% CI 10.4, 15.2) per 100 py at risk for women and 7.8 (95% CI 5.1, 10.6) per 100 py at risk for men. Women had a 1.7-fold increased risk of developing UTI compared to men (HR 1.7 (95% CI 1.1, 2.5); *P *= 0.012).

### Predictive factors of urinary tract infections

Table 1 shows the incidences of UTI in various groups for the studied predictive factors with their corresponding hazard ratios (HRs). The occurrence of UTI was univariately associated with (listed highest to lowest HR): a history of UTI between the ages of 85 and 86 years, unintentional loss of faeces, severe cognitive impairment (MMSE < 19), institutionalisation, disability in daily living, self-reported urine incontinence, stroke, gender, education, and medical diagnosis of incontinence. UTIs were not associated with income, smoking, body mass index ≥ 27, depressive symptoms, diabetes mellitus, BPH, cancer, severity of urine incontinence, pad use, creatinine clearance < 30 mL/minute and C-reactive protein > 5 mg/L.

Additional analysis showed that all single items of the GARS (walk inside, get out of bed, get into and out of a chair, use the toilet, wash hands and face, wash body, dress and undress, eat and drink, and make breakfast) predicted the risk in developing UTI (Table 2).

**Table 2:** Risk of UTI from age 86 years onwards depending on disability (n = 479)

	Index group N (%)	Incidence in Index group, per 100 py (95% CI)	Incidence in reference group*, per 100 py(95% CI)	HR (95%CI)	*P-*value
Going to the toilet	103 (21.5)	5.9 (4.5, 7.7)	2.6 (2.1, 3.2)	3.8 (2.7, 5.4)	< 0.001
Drinking and feeding oneself	44 (9.2)	6.0 (4.0, 9.0)	3.0 (2.5, 3.6)	3.5 (2.3, 5.6)	< 0.001
Washing hands and face	65 (13.6)	5.6 (4.0, 7.9)	2.9 (2.4, 3.6)	3.3 (2.2, 4.9)	< 0.001
Preparing breakfast	80 (16.7)	5.3 (3.8, 7.3)	2.9 (2.4, 3.5)	3.1 (2.1, 4.6)	< 0.001
Getting into and out of bed	128 (26.7)	5.1 (3.9, 6.6)	2.7 (2.2, 3.3)	2.6 (1.9, 3.7)	< 0.001
Getting around the house	133 (27.8)	4.6 (3.5, 6.0)	2.8 (2.2, 3.4)	2.4 (1.7, 3.3)	< 0.001
Washing whole body	204 (42.6)	4.3 (3.5, 5.4)	2.5 (2.0, 3.3)	2.3 (1.7, 3.3)	< 0.001
Dressing oneself	183 (38.2)	4.3 (3.4, 5.4)	2.7 (2.1, 3.4)	2.1 (1.5, 3.0)	< 0.001
Standing up from a chair	147 (30.7)	4.3 (3.3, 5.6)	2.9 (2.3, 3.5)	2.0 (1.4, 2.8)	< 0.001

py = person-years; CI = confidence interval; HR = hazard ratio;

* Definition of reference groups: no disability going to the toilet, no disability drinking and feeding oneself, no disability washing hands and face, no disability preparing breakfast, no disability in getting into and out of bed, no disability in getting around the house, no disability washing whole body, no disability dressing oneself, no disability standing up from chair.

After multivariate analysis, severe cognitive impairment (MMSE < 19), disability in daily living, UTI between the ages of 85 and 86 years and self-reported urine incontinence remained independently and significantly predictive for the occurrence of UTI (Table 3).

**Table 3:** Factors predictive for increased risk of developing UTI after age of 86 years onwards by multivariate Cox regression analysis and Population Attributable Risk (PAR) of the occurrence of UTI (n = 479)

	N	HR (95%CI)	PAR (%)
*Functioning*			
Severe cognitive impairment (MMSE < 19)	88	1.9 (1.3, 2.9)	19.6
Disability in daily living †	275	1.7 (1.1, 2.5)	43.8
*Co-morbidities*			
UTI between the ages of 85 and 86 years	68	3.4 (2.4, 5.0)	24.1
Self-reported urine incontinence	231	1.5 (1.0, 2.1)	33.0

Variables in multivariate Cox regression model: gender, institutionalisation, education, severe cognitive impairment (MMSE < 19), disability in daily living, UTI between the ages of 85 and 86 years, stroke, unintentional loss of faeces, self-reported urine incontinence and medical diagnosis urine incontinence.

CI = confidence interval; HR = hazard ratio; MMSE = Mini-Mental State Examination; UTI = urinary tract infection

† Disability in daily living = unable to do any one of the nine basic activities of daily living independently, according to the Groningen Activity Restriction Scale.

In both women and men, a UTI between the ages of 85 and 86 years was predictive for developing UTI from age 86 onwards (in women HR 3.8 (95% CI 2.5, 5.6); *P *< 0.001 and in men HR 4.4 (95% CI 1.8, 10.8); *P *= 0.001). Further stratified analysis showed that severe cognitive impairment (MMSE < 19) was associated with a three times higher risk in developing UTI in women (HR 3.0 (95% CI 2.0, 4.5); *P *< 0.001), but not in men (HR 1.5 (95% CI 0.6, 3.9); *P *= 0.41). Moreover, stroke showed significantly higher risk for developing UTI in women (HR 2.0 (95% CI 1.2, 3.4); *P *= 0.005), but not in men (HR 1.6 (95% CI 0.6, 4.2); *P *= 0.346). Also higher risk was found for CRP > 5 mg/l in women (HR 1.5 (95% CI 1.0, 2.2); *P *= 0.049), but not in men (HR 0.8 (95% CI 0.4, 1.8); *P *= 0.633). Stratification for living situation (independently or long-term care facility) shows no differences in predictive factors for UTI (Table 4).

**Table 4:** Predictive factors for UTI stratified for living situation by univariate Cox regression analysis after the age of 86 years

	Independently (N = 372)			Long-term care facility (N = 107)		
		
	N	HR (95%CI)	*P-*value	N	HR (95%CI)	*P-*value
*Socio-demographic factors*						
Female	242	1.4 (0.9, 2.3)	0.127	80	2.1 (0.9, 4.9)	0.098
Low income	170	1.0 (0.7, 1.5)	0.914	68	0.9 (0.5, 1.6)	0.650
Primary school only	221	1.2 (0.8, 1.9)	0.452	75	2.3 (1.1, 4.9)	0.036
Smoking (current)	55	1.1 (0.6, 1.9)	0.817	15	1.4 (0.6, 3.1)	0.443
Body Mass Index ≥27	171	1.2 (0.8, 1.8)	0.329	41	0.9 (0.4, 1.7)	0.655
*Functioning*						
Severe cognitive impairment (MMSE < 19)	36	1.9 (1.1, 3.4)	0.030	54	2.3 (1.2, 4.4)	0.009
Depressive symptoms (GDS-15 > 4)	46	1.2 (0.6, 2.2)	0.578	12	0.2 (0.0, 1.7)	0.146
Disability in daily living †	191	1.9 (1.3, 2.9)	0.002	92	3.6 (1.1, 11.7)	0.033
*Co-morbidities*						
Diabetes mellitus	53	0.8 (0.4, 1.5)	0.536	23	1.1 (0.5, 2.6)	0.759
Stroke	31	1.4 (0.8, 2.8)	0.269	29	1.6 (0.9, 3.1)	0.143
Cancer	80	1.1 (0.6, 1.8)	0.845	17	1.0 (0.4, 2.4)	0.963
Benign Prostatic Hyperplasia (IPSS score ≥8)	49	1.3 (0.6, 2.9)	0.521	4	NA	NA
UTI between the ages of 85 and 86 years	41	3.3 (2.1, 5.3)	< 0.001	31	4.1 (2.2, 7.5)	< 0.001
Unintentional loss of faeces	35	2.7 (1.6, 4.8)	< 0.001	30	2.5 (1.3, 4.6)	0.004
Self-reported urine incontinence	143	1.7 (1.2, 2.6)	0.007	69	1.9 (0.9, 4.0)	0.071
Medical diagnosis incontinence	69	1.3 (0.8, 2.2)	0.260	26	1.7 (0.9, 3.4)	0.110
PRAFAB score ≥11	76	0.7 (0.3, 1.7)	0.462	24	NA	NA
PRAFAB: Pad use	69	1.0 (0.4, 2.2)	0.949	22	1.9 (0.3, 15.1)	0.530
Creatinine clearance < 30 mL/minute	29	0.8 (0.4, 1.6)	0.487	14	1.8 (0.5, 5.8)	0.337
CRP > 5 mg/L	116	1.2 (0.8, 1.8)	0.425	43	1.1 (0.6, 2.1)	0.664

HR = hazard ratio; CI = confidence interval; CRP = C-reactive protein; GDS-15 = 'Geriatric Depression Scale' with 15 items (only administered to participants with MMSE ≥19); IPSS = 'International Prostate Symptom Score' (only administered to male participants with MMSE-score ≥19); MMSE = Mini-Mental State Examination; PRAFAB = 5-item questionnaire score incontinence (only administered to participants with MMSE-score ≥19); py = person-years; UTI = urinary tract infection.

† Disability in daily living = unable to do any one of the nine basic activities of daily living independently, according to the Groningen Activity Restriction Scale

NA = not applicable

**Figure 1:**
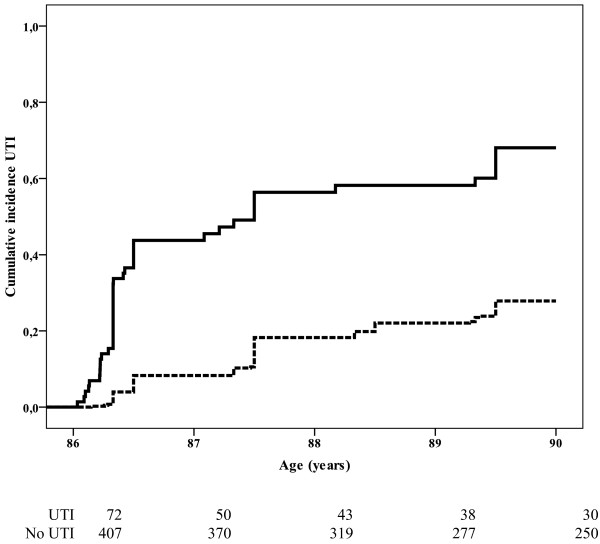
**Cumulative incidence of UTI from age 86 onwards depending on history of UTI between the age of 85 and 86 years**. Black line: participants with episode of UTI between the ages of 85 and 86 years (n = 72). Dotted line: participants without episode of UTI between the ages of 85 and 86 years (n = 407).

Participants with UTI between the ages of 85 and 86 years had an increased risk of developing UTI during follow-up compared to participants without an episode of UTI between the ages of 85 and 86 years (Figure 1 and Table 1; HR 4.1 (95% CI 2.9, 5.9)). The risk of a recurrent UTI was greatest within the first year of follow-up (HR: 6.8 (95% CI 4.1, 11.1), HR second to fourth year: 1.8 (95% CI 0.9, 3.6)).

Additional analysis showed that among participants without an UTI between the ages of 85 and 86 years, female gender and stroke were predictors of developing UTI (HR 1.5 (95% CI 1.0, 2.4); *P *= 0.059 for gender and HR 2.0 (95% CI 1.2, 3.2); *P *= 0.011 for stroke, respectively). No further differences in hazard ratios were observed for the other potentially predictive factors between participants who had had a UTI between the ages of 85 and 86 years and participants who had not had an UTI.

### Population Attributable Risk

Table 3 presents the relative contribution of the various predictive factors to the occurrence of UTI from age 86 years onwards, expressed by the Population Attributable Risk (PAR) of each variable that was shown to be predictive of UTI in the multivariate analysis. The highest PARs for the development of UTI were found for disability in daily living (44%), self-reported incontinence (33%), history of UTI between the age of 85 and 86 years (24%) and severe cognitive impairment (20%). After multivariate analysis gender, institutionalisation, education, stroke, unintentional loss of faeces and medical diagnosis urine incontinence were not predictive any more for UTI.

## Discussion

In this population-based prospective follow-up study among the oldest old, the incidence of UTI was 11.2 per 100 person years at risk. Severe cognitive impairment, disability in daily living, history of UTI between the age of 85 and 86 years and self-reported urine incontinence were among the strongest predictors for developing UTI from age 86 years onwards. The Population Attributable Risk was highest for disability in daily living (44%).

As in other studies, we found females to have a greater risk of developing UTI than men. However, in the multivariate analysis gender was no longer a predictor of UTI. Possibly, in old age, other predictors play a greater role in predicting UTI than gender *per se*. In spite of the differences between older persons living independently and older persons living in long-term care facilities; we found the same predictive factors for UTI from the age 86 years onwards in both populations.

It is well known that patients with a history of UTI have a higher rate of UTI than those without a history of UTI [1,20]. In our study a history of UTI between the ages of 85 and 86 years was a strong predictor of recurrent UTI from the age of 86 years onwards. Besides a history of UTI between the age of 85 and 86 years, our study showed that disability in daily living and severe cognitive impairment, both factors reflecting declined functional status, were also predictors of UTI. These findings are in line with other studies [16,19,20]. Since 19% of our population-based sample was severely cognitively impaired and almost 60% had disability in daily activities, declined functional status greatly contributes to the occurrence of UTI in the general oldest old population.

Although diabetes mellitus has been shown to be associated with greater predisposition to UTI in other study populations [17,18,37-39], we did not find any association between diabetes mellitus and UTI in our study population. Perhaps differences in age, type of diabetes and definition of UTI explain these contradictory findings. On the other hand, previous studies showed that diabetes was no longer associated with cognitive decline and the incidence of lower respiratory tract infections at old age, indicating that the clinical impact of diabetes is possibly diminished in old age [40,41].

The present study is a unique population-based sample of participants aged 86 years and over, with extensive baseline measurement and almost complete follow-up for morbidity and mortality. To our knowledge, we are the first to examine the incidence and predictive factors of UTI in a large group of unselected very old individuals in a population-based setting. It is important to study predictive factors of UTI in old age specifically, because they are the fastest growing part of the general population and the incidence of UTI increases with age [3-5]. The fact that we only studied 86-year-olds could also be considered a limitation of our study. Since bladder structure and function, and the immune system have been shown to change with age [3], our results may not be generalized to younger elderly. Another limitation of our study might be that UTIs were diagnosed during clinical practice, not diagnosed by standardised diagnostic study procedures. In our study, however, all UTIs were diagnosed by general practitioners and elderly care physicians based on signs and symptoms and urine analysis. This procedure reflects usual care and enables generalisation of our results to daily care for the oldest old.

It is generally known that symptomatic UTI is over-diagnosed in elderly populations given the high prevalence of asymptomatic bacteriuria [5], especially in long-term care facilities with a prevalence of 25 to 50% [22]. Also difficulties in communication, chronic genitourinary symptoms, and the high frequency of positive urine cultures, make ascertainment of symptomatic UTI problematic for the functionally impaired elderly [22]. Also urine cultures are often contaminated and the lack of existence of specific markers of infectious bladder inflammation makes it difficult to diagnose UTI in impaired elderly. In our study, we found an UTI incidence of 11.2 per 100 person years at risk for persons aged 86 years and over. This may be an overestimation due to the presence of bacteriuria. However, the incidence found in this study is comparable with data from the Dutch national GP registration (11.7 for men and 29.4 for women aged 85 years and over) [4] and the fact participants actively visited their treating physician with UTI like symptoms.

Further, it is known that long-term catheterization is a strong risk factor for UTI and bacteriuria in institutionalized older persons [10]. Unfortunately, we did not have any information about long-term catheterization in our study population and could not report on the predicting effect. These is a limitation of our study, but we believe that this may not have affected the results of our study much, since in Dutch nursing homes there is a policy to avoid the use of catheters *in situ *and it has been so for many years [42].

## Conclusions

In older populations, UTIs account for nearly 25% of all infections [1,2]. As UTIs are associated with serious negative outcomes, it is important to consider preventive strategies for UTI in older individuals. Our study showed four important predictors for UTI: severe cognitive impairment, disability in ADL, history of UTI between the ages of 85 and 86 years, and self-reported urine incontinence. Remarkably, none of these predictors appear to be modifiable. However, these predictors could still be used in the development of a clinical prediction rule to select for whom apply preventive strategies. Prophylaxis with low-dose, long-term antibiotics [12,13], estrogens [14] and cranberry products [12,15] are potential strategies to prevent UTI, but so far none of these strategies have been proven to prevent UTI in the very old. Selection of high risk oldest old is a crucial first step in successful prevention of UTI. Before these preventive strategies may be introduced in the oldest old, their effects and side effects have to be studied in randomised intervention studies.

## Abbreviations

ADL: activities of daily living; BMI: body mass index; BPH: prostatic hyperplasia; CI: confidence interval; CRP: C-reactive protein; GARS: Groningen Activity Restriction Scale; GDS: Geriatric Depression Scale; HR: hazard ratio; ICD: international classification of diseases and related disorders; IPSS: International Prostate Symptom Score; LUTS: lower urinary tract symptoms; MMSE: Mini-Mental State Examination; PAR: Population Attributable Risk; py: person-year; UTI: urinary tract infection.

## Competing interests

The authors declare that they have no competing interests.

## Authors' contributions

MC and WE contributed to the analysis and interpretation of the data, drafting of the manuscript, critical revision of the manuscript and statistical analysis. HC contributed to the interpretation of the data, drafting of the manuscript and critical revision of the manuscript. JG contributed to the study concept and design, acquisition of data, analysis and interpretation of the data, drafting of the manuscript and critical revision of the manuscript. All authors read and approved the final version of the manuscript.

## Pre-publication history

The pre-publication history for this paper can be accessed here:

http://www.biomedcentral.com/1741-7015/9/57/prepub
